# Health-Promoting Potential of Mandarin Pomace Extracts Enriched with Phenolic Compounds

**DOI:** 10.3390/nu16142370

**Published:** 2024-07-22

**Authors:** Adriana Maite Fernández-Fernández, Eduardo Dellacassa, Romina Curbelo, Tiziana Nardin, Roberto Larcher, Alejandra Medrano-Fernandez, María Dolores del Castillo

**Affiliations:** 1 Departamento de Ciencia y Tecnología de Alimentos, Facultad de Química, Universidad de la República, General Flores 2124, Montevideo 11800, Uruguay; afernandez@fq.edu.uy (A.M.F.-F.); amedrano@fq.edu.uy (A.M.-F.); 2 Instituto de Investigación en Ciencias de la Alimentación (CIAL) (CSIC-UAM), C/Nicolás Cabrera, 9, Campus de la Universidad Autónoma de Madrid, 28049 Madrid, Spain; 3 Graduate Program in Chemistry, Facultad de Química, Universidad de la República, General Flores 2124, Montevideo 11800, Uruguay; 4 Departamento de Química Orgánica, Facultad de Química, Universidad de la República, General Flores 2124, Montevideo 11800, Uruguay; edellac@fq.edu.uy (E.D.); rcurbelo@fq.edu.uy (R.C.); 5 Dipartimento Alimenti e Trasformazione, Centro Trasferimento Tecnologico, Fondazione Edmund Mach di San Michele all’Adige, Via E. Mach, 1 38010 Trento, Italy; tiziana.nardin@fmach.it (T.N.); roberto.larcher@fmach.it (R.L.)

**Keywords:** α-amylase, anti-inflammatory, antioxidant, bioaccessibility, chronic diseases, flavonoids, hydro-alcoholic-acid extracts, mandarin pomace, α-glucosidase, phenolic compounds

## Abstract

The aim of this work was to assess the effect of in vitro human digestion on the chemical composition (carbohydrates and phenolic compounds) and bioactivity of hydro-alcoholic-acid pomace extracts from two mandarin varieties (Clemenule and Ortanique) by measuring their antioxidant, antidiabetic, anti-glycative, hypolipidemic, and anti-inflammatory properties. The phenolic compound profile showed that nobiletin was the main flavonoid found in the extracts and digests of Clemenule pomace and extract, while isosinensetin/sinensetin/tangeretin were the ones in the Ortanique samples. The digests of Clemenule and Ortanique extracts showed Folin reaction values of 9.74 and 9.20 mg gallic acid equivalents (GAE)/g of sample, ABTS values of 83.2 and 91.7 µmol Trolox equivalents (TE)/g of sample, and ORAC-FL values of 142.8 and 891.6 µmol TE/g of sample, respectively. Extracts (50–500 µg/mL) inhibited intracellular reactive oxygen species (ROS) formation in CCD-18Co cells under physiological and oxidative-induced conditions. Clemenule and Ortanique extract digests showed IC_50_ values of 13.50 and 11.07 mg/mL for α-glucosidase, 28.79 and 69.64 mg/mL for α-amylase, and 16.50 and 12.77 mg/mL for AGEs, and 2.259 ± 0.267 and 0.713 ± 0.065 mg/mL for pancreatic lipase inhibition, respectively. Ortanique extract (250–1000 µg/mL) inhibited the production of nitric oxide in RAW264.7 macrophages under inflammation-induced conditions, and intracellular ROS formation. In conclusion, altogether, the results supported the potential of mandarin extracts to be used as health promoters by reducing the risk of non-communicable chronic diseases.

## 1. Introduction

One of the most recent trends in the agri-food industry is the utilization of byproducts to apply the circular economy concept by developing value-added products [1]. The citrus industry generates large amounts of byproducts during juice production (half of citrus fruit weight), mainly peels, seeds, and remaining pulp from juice elaboration, also called pomace, thus wasting a rich source of bioactive compounds, specifically phenolic compounds [2] and carotenoids [3]. Nobiletin, heptamethoxyflavone, hesperidin/neohesperidin, tangeretin, tetramethylscutellarein, and naringin/narirutin are the main flavonoids present in the phenolic portion of mandarin pomace from Clemenule and Ortanique varieties [4], representing a potential source of bioactive compounds for the prevention/treatment of metabolic disorders through its use as functional ingredients [5].

Some non-communicable chronic diseases are associated with metabolic disorders, which significantly reduce the quality of life for those affected. Type 2 diabetes exemplifies this, causing numerous complications which include nephropathy, neuropathy, retinopathy, and a compromised immune system, among others [6]. Insulin resistance is a characteristic of type 2 diabetes and obesity that causes intracellular oxidative stress [6] (implicated in many chronic diseases), resulting in excessive reactive oxygen species (ROS) production, which leads to apoptotic or necrotic cell death [7]. ROS release may come from activated macrophages and neutrophils during inflammatory damage, leading to inflammation propagation by stimulating cytokine release and recruiting additional immune cells [8]. Thus, controlling the excessive production of ROS could mitigate inflammation through mandarin pomace compound consumption.

Another feature of type 2 diabetes is persistent hyperglycemia, which could be controlled by inhibiting carbohydrases during digestion (such as α-amylase and α-glucosidase) to slow glucose absorption and reduce post-prandial blood glucose peak [9]. Furthermore, hyperglycemia promotes protein glycation, generating advanced glycation end products (AGEs) in tissues, which increase the release of free radicals and pro-inflammatory molecules [10,11]. Type 2 diabetes, among other chronic diseases (also hypertension, mental health disorders, cardiovascular diseases, and certain cancers) includes obesity as a risk factor. Obesity comprises excessive body fat accumulation and macrophage infiltration in adipose tissue, causing inflammation [12]. In this sense, pancreatic lipase inhibition could contribute to fat accumulation control as participates in triglyceride absorption through the release of monoacylglycerides and free fatty acids by hydrolysis [12]. Excessive energy consumption causes the subsequent attraction of immune cells like macrophages, triggering chronic inflammation. Therefore, identifying anti-inflammatory compounds, such as nobiletin, naringenin, naringin, hesperidin, and tangeretin [13], is pivotal for its prevention and complication avoidance in these conditions.

However, their health-promoting effects depend on their bioaccessibility and bioavailability, with the former representing the bioactive compounds that are released from the food matrix and which obtain the bioaccessible fraction [14] and can be potentially absorbed in the intestine. The bioactive compounds may be degraded/transformed under digestion conditions. In vitro studies carried out by others support this fact [9,14]. In addition, the chemical structure of phenolic compounds plays a pivotal role in their stability during digestion, leading to changes in bioactive properties [15]. Thus, the identification of the phenolic compounds and the determination of bioactive properties after digestion (bioaccessibility studies) are of great importance to estimate if the remaining phenolic compounds are able to exert bioactive properties.

The present investigation aimed to assess the potential of mandarin pomace hydro-alcoholic-acid extracts for reducing the risk of metabolic disorders such as type 2 diabetes. To achieve this goal, the extracts’ phenolic composition and in vitro bioactivity before and after the *in vitro* simulation of human digestion was measured to gain an insight on their potential prophylactic and therapeutic effects as well as the mechanism of action of their bioactive compounds.

## 2. Materials and Methods

**Figure 1:**
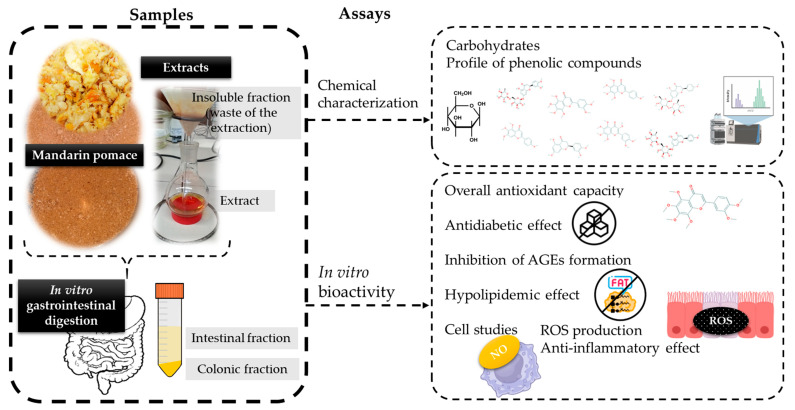
Scheme of the research carried out in the current work.

Figure 1 shows a scheme of the obtained samples and assays carried out in the present research to better understand it.

### 2.1. Chemical Reagents

All the reagents used in the present work were of reagent grade; the following standards and enzymes were purchased from Sigma-Aldrich (St. Louis, MO, USA): gallic acid; 6-hydroxy-2,5,7,8-tetramethylchroman-2-carboxylic acid (Trolox); acarbose; Folin reagent; 2,20-azinobis-(3-ethylbenzothiazoline-6-sulfonic acid); diammonium salt (ABTS); 2,20-azobis (2-methylpropionamidinedihydrochloride (AAPH); fluorescein disodium salt (FL); α-glucosidase from rat intestine acetone powder; human α-amylase; pancreatic lipase; 4-methylumbelliferyl-α-D-glucopyranoside; 4-methylumbelliferyl oleate (4-MUO); starch; and 3,5-dinitrosalicylic acid. Potassium persulphate was purchased from J. T. Baker, and Orlistat standard was purchased from Alfa Aesar (Haverhill, MA, USA). Megazyme K-SUFRG test kit (Chicago, IL, USA) was used for sugar (sucrose, fructose, and glucose) determination.

Normal human colon cell line (CCD-18Co) and mouse macrophage cells (RAW 264.7) from American Type Culture Collection (ATCC, Manassas, VA, USA) were used for cell culture studies. Complete cell culture medium included L-glutamine (1%, *v*/*v*) and antibiotics (penicillin and streptomycin 1:1, 1% *v*/*v*) in Dulbecco’s modified Eagle medium (DMEM) (Gibco Laboratory, Invitrogen Co., Grand Island, NY, USA) and heat-inactivated fetal bovine serum (FBS, 10% *v*/*v*, Hyclone, GE Healthcare, Chicago, IL, USA). Trypsin was purchased from Gibco Laboratory (Invitrogen Co., Grand Island, NY, USA). Cell culture reagents and standards were purchased from Sigma-Aldrich (St. Louis, MO, USA): ascorbic acid, sodium nitrite, 2′,7′-dichlorofluorescin diacetate (DCFH-DA), 3-(4,5-dimethylthiazol-2-yl)-2,5-diphenyltetrazolium bromine (MTT), tert-butyl hydroperoxide, sulfanilamide, N-(1-napthyl) ethylenediamine dihydrochloride, phosphoric acid, and lipopolysaccharide (LPS) from *E. coli* O55:B5.

### 2.2. Samples

#### 2.2.1. Raw Material

A wet mixture of pulp, peel, and seeds (pomace) from Clemenule and Ortanique mandarin (*Citrus reticulata*) were obtained from Azucitrus (Paysandú, Uruguay).

#### 2.2.2. Mandarin Pomace Extracts

The solvents used for extraction were chosen based on the solubility and stability of the phenolic compounds, especially flavonoids, present in citrus pomaces [16]. The extraction was performed using methanol/water/formic acid (70:25:5) as previously described [17]. After the extraction process, solvents were evaporated, the solid residue was re-constituted in water, freeze-dried, and stored at −20 °C for subsequent analysis. The waste of the extraction was dried in an oven at 40 °C for 24 h (up to constant weight) and stored at −20 °C for subsequent analysis. According to the Directive 2009/32/EC (https://eur-lex.europa.eu/legal-content/EN/TXT/?uri=CELEX:02009L0032-20100916 (accessed on 29 June 2024)), the use of methanol is authorized with a maximum residue limit in the extracted foodstuff or food ingredient of 10 mg/kg. In the case of the present work, the solvents were evaporated after the extraction and re-dissolved in water. However, studies regarding toxicity and levels of methanol in the extracts should be addressed to ensure food safety.

#### 2.2.3. In Vitro Human Digests

The Hollebeeck, Borlon, Schneider, Larondelle, and Rogez [18] digestion model was performed to mimic human digestion. After a duodenal step of the digestion, samples were heated in a water bath at 90 °C for 10 min and then centrifuged (10,000 rpm for 10 min). The soluble (supernatant) and insoluble (pellet) fractions, also called intestinal and colonic fractions, respectively, were freeze-dried and stored at −20 °C for further analysis. HPLC-DAD-MS was performed to evaluate the composition, and bioactivity assays were carried out on digestion samples.

### 2.3. Chemical Characterization of Dried Mandarin Pomace and Extracts

- Carbohydrates

The phenol–sulphuric method, as described by Masuko et al. [19] with some modifications [20], was used for determining the total carbohydrate content in the pomace, extracts, and insoluble fractions (waste of the extraction process). Sugar (fructose, glucose and sucrose) content was determined in the pomace, extracts, and insoluble fractions (waste of the extraction process), as well as in the intestinal fractions of pomaces and extracts, by a standard commercial enzymatic method using the Megazyme K-SFG kit (Megazyme, Chicago, IL, USA). Results were expressed as g of sugar/100 g of dry sample.

- Profile of phenolic compounds

The phenolic profile of Clemenule and Ortanique pomace extracts (10 mg/mL in H_2_O:MeOH, 50:50, *v*/*v*) was performed via UHPLC-MS (Thermo Ultimate R3000 UHPLC, Thermo Scientific, Sunnyvale, CA, USA) as described by Fernández-Fernández et al. [4]. The detection was assessed with a QExactive TM hybrid quadrupole–orbitrap mass spectrometer (HQ-OMS, Thermo Scientific, Bremen, Germany) under heated electrospray ionization (HESI-II), in negative ion mode.

The identification of phenolic compounds in the intestinal and colonic fractions of mandarin pomace and extracts was performed as described by Olt et al. [21] with some modifications. The freeze-dried samples were prepared in H_2_O:MeOH (50:50, *v*/*v*). Briefly, an HPLC Kinetex C18-EVO was used (C18 column, 5 µm particle size, 150 × 4.6 mm i.d., Phenomenex, CA, USA) at 35 °C with a Shimadzu Triple Quadrupole MS detector (Shimadzu, Tokyo, Japan) with an electrospray ionization (ESI) interface. The gradient of the mobile phase at a flow rate of 1.3 mL/min was 0–100% A (0.1% trifluoroacetic acid) for 3 min, 4–30% B (acetonitrile) for 50 min, 30–98% B for 5 min, and isocratic 98% B for 2 min. The detection was performed at 280 nm.

### 2.4. In Vitro Bioactivity

Overall antioxidant capacity was determined on mandarin pomaces, extracts, and their intestinal fractions, as well as mandarin insoluble fractions (waste from the extraction process). Antidiabetic effect and the inhibition of AGE formation were assessed on mandarin pomaces, extracts, and their intestinal fractions, while hypolipidemic effect was determined on mandarin pomace and extracts. Cell studies were assessed on extracts (before in vitro simulation of digestion).

- Overall antioxidant capacity

Total polyphenol content (TPC) was performed by the Folin–Ciocalteu method as described by Slinkard and Singleton [22] with some modifications [20]. Samples were prepared according to Fernández-Fernández et al. [20]. Results were expressed as g gallic acid equivalents (GAE)/100 g of sample. The calibration curve of standard gallic acid (0.05–1.0 mg/mL) was used for the quantitative analysis.

ABTS assay was performed as described by Re et al. [23] with some modifications [20]. Results were expressed as μmol Trolox equivalents (TE)/g of sample. Trolox calibration curve (0.25–1.5 mM) was used for the quantitative analysis.

ORAC-FL assay was performed as previously described [24,25]. Duplicate preparation of sample and at least a triplicate of analysis were conducted.

Trolox calibration curve (10–80 µM) was constructed and results were expressed as μmol TE/g of sample.

- Antidiabetic effect

α-Glucosidase and α-amylase inhibition assays were performed according to Fernández-Fernández et al. [20] and Li, Yao, Du, Deng, and Li [26] with some modifications [17], respectively. Acarbose was used as the control in both assays. Fluorescence (λex = 360 ± 40 nm and λem = 460 ± 40 nm) was measured in a Varioskan Lux (Thermo Scientific) fluorimeter microplate reader at 37 °C. Sample concentrations ranging from 0.25 to 15.0 mg/mL were analyzed in α-glucosidase assay to obtain IC_50_ values (mg/mL) by interpolation of the 50% of inhibition in a logarithmic function (y = aLnx + b, R^2^ = 0.98). In addition, for α-amylase inhibition, a colorimetric assay with dinitrosalicylic acid color reagent was performed. The measures were carried out at 540 nm in a Varioskan Lux (Thermo Scientific) microplate reader. Sample concentrations ranging from 2.5 to 150.0 mg/mL were analyzed in order to obtain IC_50_ values (mg/mL) by interpolation of the 50% of inhibition in a logarithmic function (y = aLnx + b, R^2^ = 0.98). Both analyses were performed in quadruplicate.

- Inhibition of AGE formation

The analysis was performed as described by Starowicz and Zieliński [27]. Fluorescent AGEs were formed using reacting bovine serum albumin (BSA, 1 mg/mL) and methylglyoxal (MGO, 5 mM) in PBS 10 mM pH 7.4 with 0.02% sodium azide without (positive control) and with samples at 37 °C for 7 days. The pharmaceutical of reference aminoguanidine (AG) was used in a range of concentrations of 1.0 to 8.0 mM, and samples were tested at concentrations of 0.25–50 mg/mL for IC_50_ value calculation. Fluorescence was measured (λ_excitation_ = 340 nm, λ_emission_ = 420 nm) in a Varioskan Lux (Thermo Scientific) fluorimeter microplate reader. IC_50_ values were obtained by interpolation of the 50% of inhibition in a logarithmic function (y = aLnx + b, R^2^ = 0.98). Analysis was performed in quadruplicate. Inhibition percentage was calculated as follows:

$$\%\;AGEs\;Inhibition=\frac{F_{C+}-\left(F_{sample}-F_{blank}\right)}{F_{C+}}\times 100$$

where positive control is the fluorescence measurement *F_C_*_+_, fluorescent measurement of different concentrations of the samples is *F_sample_*, and fluorescent measurement of sample blank is *F_blank_*.

- Hypolipidemic effect

Pancreatic lipase inhibition assay was carried out by testing samples (0.25–50 mg/mL), phenolic compounds’ standards [gallic acid (Ga), chlorogenic acid (Cl), caffeic acid (Ca), and rutin (Ru)] and Orlistat (pharmaceutical of reference) as described by Fernández-Fernández et al. [20]. Fluorescence was measured in a Varioskan Lux (Thermo Scientific) fluorimeter microplate reader (λ_excitation_ = 360 ± 40 nm, λ_emission_ = 460 ± 40 nm) after 30 min of incubation at 25 °C. IC_50_ values were obtained by the interpolation of the 50% of inhibition in a logarithmic function (y = aLnx + b, R^2^ = 0.98). Analysis was performed in quadruplicate.

- Cell studies
  Normal human subepithelial myofibroblast colonic cells (CCD-18Co) and mouse macrophages (RAW264.7) were used. Cells were grown using 75 cm^2^ cell culture flasks in complete cell culture medium (described in Section 2.1), incubating at 37 °C and 5% CO_2_ (100% relative humidity) for 24 h till cell confluence was achieved. The extract tests were performed in triplicate and in three different passages by preparing 10 mg/mL of solutions in PBS (10 mM; pH 7.4) and filtered with a porous medium of 0.22 µm. MTT assay was carried out to determine the cell viability of different concentrations of mandarin extracts [4] (see Supplementary Materials).

Reactive Oxygen Species (ROS) production assay
  CCD-18Co and RAW 264.7 cell ROS production was measured [28] by seeding 10,000 CCD-18Co and 80,000 RAW264.7 cells/well. DCFH-DA fluorescent probe was employed to measure ROS within the cell by performing the following steps: add different concentrations of the extracts (1–1000 µg/mL) to each well; perform incubation for 24 h; add 2 µL of DCFH-DA to each well; perform incubation for 30 min; remove supernatants; perform one cell wash with PBS; and add the same extract solutions to each well and measure fluorescence in a microplate reader at λ_excitation_ = 485 nm and λ_emission_ = 528 nm. Under oxidation-induced conditions, tert-butyl hydroperoxide (1 mM) was used as the oxidative agent. Afterwards, cell viability was assessed (20 µL of MTT reagent in each well), following the described procedure in the Supplementary Materials, to correct ROS values with cell viability:
  $$\%\;ROS=\frac{Fluorescence_{sample}}{Absorbance_{MTT\;sample}}*\frac{Absorbance_{MTT\;C-}}{Fluorescence_{C-}}*100$$
Anti-inflammatory effect
  Nitric oxide (NO) production was measured in RAW264.7 mouse macrophages under stimulating conditions (lipopolysaccharide, LPS) according to Fernández-Fernández et al. [20]. The macrophages on each well were treated with concentrations of mandarin extracts (250–1000 µg/mL) prepared in a medium without FBS and incubated for 24 h, followed by a transfer of cells’ supernatants to another 96-well plate. Then, Griess reagent was added to each well, incubation was performed for 15 min at room temperature, and measurement was taken at 550 nm in a microplate reader. In parallel, the standard curve (sodium nitrite, 0–10 µg/mL) was also measured. Positive and negative controls were also measured for a medium without FBS but with LPS, and a medium without FBS, respectively.

### 2.5. Statistical Analysis

Analysis of variance (ANOVA) was performed, followed by the Tukey test and LSD Fisher test (cell studies) to determine significant differences between values (*p* < 0.05) using Infostat v. 2015 program.

## 3. Results and Discussion

### 3.1. Composition on Carbohydrates

Table 1 shows the content of total carbohydrates and sugars found in raw mandarin pomaces, their extracts, and the waste of the extraction process (insoluble fraction). The extracts showed double the value of total carbohydrate content compared to the raw byproduct content, meaning that the extraction favors the simple carbohydrate release. Accordingly, sucrose and fructose contents in the extracts were higher, while glucose was only higher for the Ortanique extract (*p* < 0.05). In general, mandarin byproducts’ insoluble fractions presented the lowest values of carbohydrates, supporting the high degree of extractability of the carbohydrate under the conditions employed in the present investigation.

Sugar contents after the in vitro simulation of digestion (Table 2) were also evaluated in order to state their effective concentration in the intestinal fraction; the results showed that sucrose and fructose values were significantly lower in pomace digests than in those corresponding to the extracts (*p* < 0.05).

### 3.2. Phenolic Profile of Mandarin Pomace Extracts

The LC-MS results of extracts showed that the flavonoid found in the highest proportion is nobiletin (Table 3). Other main flavonoids were isosinensetin/sinensetin/tangeretin, heptamethoxyflavone, tetramethylscutellarein, hesperidin/neohesperidin, and naringin/narirutin. Most of the flavonoids identified were found in greater proportion in the Ortanique extract than in Clemenule extract, except for heptamethoxyflavone, eriocitrin/neoeriocitrin, and hesperidin/neohesperidin. The results are in agreement with our previous findings on Clemenule and Ortanique pomaces [4], presenting the same tendency. However, the extracts presented concentrated proportions of some flavonoids when compared to their respective pomaces: rhoifolin/isorhoifolin, isosinensetin/sinensetin/tangeretin, nobiletin, heptamethoxyflavone, and tetramethylscutellarein. In contrast, some flavonoids (mainly from negative ESI) were found in greater proportion in mandarin pomaces: nariturin-4-glucoside/naringin glucoside, rutin in Clemenule extract, eriocitrin/neoeriocitrin in Ortanique extract, naringin/narirutin, diosmin isomer 1, and hesperidin/neohesperidin [4]. Some of these phenolic compounds were also identified on methanol extracts from sweet orange (*Citrus sinensis*) [29]. Moreover, according to Cilla et al. [30], Clemenule mandarin juices present hesperidin, narirutin, naringenin-7-O-rutinoside-4’-O-glucoside, eriocitrin, rutin, apigenin-6,8-di-C-glucoside, and dydimin in a descendent order of content, wherein some of these compounds were identified in the Clemenule extract.

The results obtained here are in agreement with those for flavonoids in citrus fruit peels, specially loose-skin mandarins, which presented mainly nobiletin and tangeretin, as in the present work [5]. Also, the current results are in agreement with the composition of a polymethoxyflavone-rich fraction from Ougan fruit (*Citrus reticulate* cv. Suavissima) that presented nobiletin and tangeretin among the top three compounds, showing frequent isomerization among sinensetin, isosinensetin, and tangeretin, which makes individual identification difficult [31].

Comparing the samples after in vitro simulation of digestion, the intestinal fraction of mandarin pomaces and their respective extracts presented the same tendency regarding the main compounds (Table 4). Nobiletin was the main compound present in the intestinal fraction of Clemenule pomace and extract in a similar proportion (74 and 78%, respectively). In the case of the intestinal fraction of Ortanique pomace and extract, the main compound was isosinensetin/sinensetin/tangeretin 1 in a similar proportion (73 and 79%, respectively). The identified phenolic compounds were found in higher content (related to a higher response) in the intestinal fraction corresponding to extracts than in those of pomaces. Differences may be due to the presence of dietary fiber in mandarin pomaces. Danero et al. [32] found that a mix of citrus flavanones (eriocitrin, neoeriocitrin, hesperidin, neohesperidin, and hesperetin) did not change after in vitro simulation of digestion.

In the case of colonic fractions, Clemenule pomace seems to retain isosinensetin/sinensetin/tangeretin 1 (64%) during digestion as it was barely present in the intestinal fraction and mostly found in the colonic fraction. In contrast, Ortanique pomace seems to retain mostly isosinensetin/sinensetin/tangeretin 3 (64.6%) in the colonic fraction and part of isosinensetin/sinensetin/tangeretin 1 (29.5%) that was not found in the intestinal fraction. Many bioactive compounds have shown a limited absorption capability thus reaching the large intestine, where they can be metabolized by microbiota [33]. Thus, the compounds that were retained in the colonic fraction may eventually have a positive effect on gut microbiota [31].

Furthermore, many flavonoids of mandarin (*Citrus reticulate*) pericarpium [34] have been found in rat plasma after ingestion, confirming their bioavailability, which may suggest that the compounds identified in the present work could follow the same tendency. Moreover, the flavonoids present in Clemenule and Ortanique extracts have shown antioxidant, antidiabetic, and anti-inflammatory properties [2].

### 3.3. Health-Promoting Potential

#### 3.3.1. Antioxidant Effect

Total antioxidant properties were determined (Table 5), highlighting that the highest Folin values corresponded to both extracts (*p* < 0.05), followed by the pomaces. The values are in agreement with those from orange pomace [4] and are higher than other mandarin peel extracts [35]. Wastes of the extraction process (insoluble fractions) presented the lowest values, reinforcing the fact that polyphenols are mostly extracted by acidified and organic solvents [20]. Red and blonde peel oranges’ extracts have recently been used in the formulation of functional gummies with remarkable antioxidant properties [36], showing the potential of citrus pomace extracts in the formulation of functional foods.

ABTS results showed the same tendency as Folin, where the extracts presented the highest antioxidant capacity (*p* < 0.05). Interestingly, ORAC-FL results showed insoluble fractions as the samples with the highest antioxidant capacity (*p* < 0.05). The main contributors to antioxidant capacity may be the phenolic compounds as reported by Y. Wang et al. [37] who studied *Citrus reticulata* Blanco varieties.

Folin values increased by the extraction but decreased during digestion. No significant differences (*p* > 0.05) were found for pomaces’ digests and their respective extracts, showing the protective effect of pomace matrix against digestion conditions. ABTS results showed the same trend as Folin values. ORAC-FL results showed a different behavior, where the digest (intestinal fraction) of Clemenule pomace and extract presented no significant differences (*p* > 0.05) in contrast with Ortanique, whose extract digest presented the highest antioxidant capacity. These results are in agreement with those reported by Li et al. [15] on the increase in ORAC-FL values of phenolic compounds after simulation of digestion.

The degradation of phenolic compounds is influenced by the phenolic compound structure [15]. The results of the present work related to antioxidant loss during digestion are in line with those from other food matrices such as red grape skin [28] and its hydro-alcoholic-acid extract [17] and Brazilian fruit purees [14], among others. Particularly, orange pomace digests have been reported to decrease the antioxidant capacity by ABTS after the digestion process [4], as well as orange peel by Folin reaction, total flavonoid content, and antioxidant capacity (CUPRAC, DPPH, and FRAP assays) [38]. Flavonoids and antioxidant activity from citrus peel extracts have shown sensitivity to pH digestion conditions [39]. Accordingly, the digest of pomegranate peel extract have also shown flavonoids and anthocyanins’ loss of stability [33]. In the same line, a study on the stability of the chemical structure and antioxidant capacity of 27 phenolic compounds reported an antioxidant capacity decrease after in vitro simulated digestion [15]. The latter decrease could be due to the intestine conditions (alkaline pH) and interactions with digestive enzymes, or to the change in the chemical structure of the initial phenolic compounds [15,35]. In contrast, some authors have reported the release of phenolic compounds from the food matrix during digestion as well as the depolymerization of phenolic compounds such as procyanidins, proanthocyanidins, and ellagitannins [40,41,42]. Nevertheless, mandarin peel extracts added to wheat bread showed a presence of the remaining phenolic compounds after in vitro simulation of digestion, with hesperidin being one of the latter [35]. All of the above supports the great potential of mandarin pomace extracts as functional ingredients. However, the extracts could not only be used as functional ingredients but could also be used as preservatives and natural food colorants [1].

**Figure 2:**
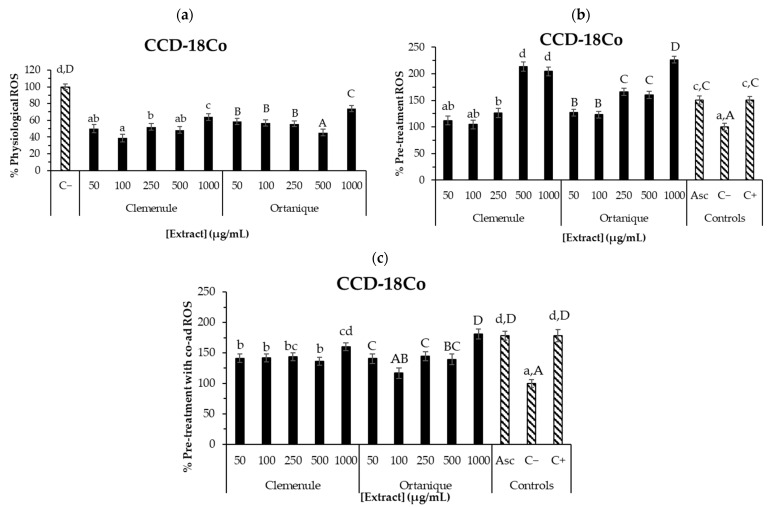
ROS production on CCD-18Co colon cells by undigested Ortanique and Clemenule extracts (**a**–**c**). Physiological ROS: 24 h incubation of cells with the samples (**a**). Pre-treatment ROS: 24 h incubation of cells with the samples, then 30 min incubation with only t-BOOH (**b**). Pre-treatment with co-ad ROS: 24 h incubation of cells with the samples, then 30 min incubation with samples and t-BOOH (**c**). Ascorbic acid (Asc, 10 µg/mL) was tested as an antioxidant of reference. Bars represent the mean values and error bars represent the standard error. Lowercase letters indicate significant differences between the samples that present them (LSD Fisher, *p* < 0.05). Capital letters indicate significant differences between the samples that present them (LSD Fisher, *p* < 0.05). All determinations were performed in triplicate in three different cell passages.

Figure 2 on CCD-18Co human normal colon cells suggests that, except for 1000 μg/mL of Ortanique extract, all the tested concentrations significantly reduced physiological ROS formation (*p* < 0.05), suggesting their potential to protect the intestinal cells against oxidative damage. In line with the present results, in a previous work, the compounds present in the intestinal fraction of the digests of Clemenule and Ortanique mandarin pomaces showed reduced ROS levels in CCD-18Co cells under tert-butyl hydroperoxide (t-BOOH, 1 mM)-induced conditions when compared to the positive control (*p* < 0.05) [4], highlighting the potential of citrus phenolic compounds on intracellular ROS formation. A similar food matrix such as tangerine (*Citrus unshiu* Marc.) pomace has shown to reduce intracellular ROS formation in Vero cells under AAPH-induced conditions [43].

Physiological and LPS-stimulated ROS levels tested in other food matrixes such as açai have shown to decrease CCD-18Co cells, finding a non-dose-dependent behavior [8] such as the one in the present work. Particularly, a hydro-alcoholic-acid extract (such as the ones in the current work) from the skin of Tannat grape pomace decreased intracellular ROS formation in CCD-18Co cells under physiological and t-BOOH-induced conditions in the range of tested concentrations (100–1000 μg/mL) [17]. Moreover, the compounds found in the intestinal fraction of the latter extract also presented decreased intracellular ROS formation in the same cell line in the pre-treatment with co-administration assay, when compared to the positive control (100–500 μg/mL).

These results suggest the antioxidant potential of Clemenule and Ortanique extracts in counteracting intracellular ROS formation on human normal colon cells (CCD-18Co) under physiological and induced conditions. Thus, these extracts could be considered to be used as functional ingredients to ameliorate oxidative stress, which may subsequently reduce the incidence of colon cancer, at least to some extent. The difference between the samples on the intracellular antioxidant effect may be due to the different phenolic profiles, considering that aglycones may have easier access into the cells and thus exert their intracellular effect [15]. Cell experiments and analysis of each concentration of the sample were performed in triplicate. The error of the measurements was of the same order of magnitude described by others in similar conditions [8]. In vivo studies should be carried out to confirm the preliminary extracts’ effects described herein. Previous reports supported this bioactivity for the compounds found in the extracts [44].

#### 3.3.2. Antidiabetic and Anti-Glycative Effect

The inhibition capacity of α-glucosidase, α-amylase, and AGEs from Clemenule and Ortanique pomaces and their extracts were assessed (Table 6) along with their positive standards. α-Glucosidase and α-amylase acarbose IC_50_ values [17] and aminoguanidine AGE-formation IC_50_ value presented significant differences with the samples in the same assay (*p* < 0.05. Clemenule and Ortanique extracts presented higher inhibition capacities (α-glucosidase, α-amylase, and AGEs; lower IC_50_ values) than their respective pomaces, which may be associated with the total polyphenol content.

α-Glucosidase plays a crucial role in carbohydrate absorption by hydrolyzing complex carbohydrates such as starch. Inhibiting its activity may represent a valuable strategy to delay post-prandial increase in blood glucose, thereby aiding in the management of type 2 diabetes [45,46,47]. Human α-amylase is primarily secreted by the pancreas and salivary glands, whereas α-glucosidase is located on intestinal cells’ membrane, specifically on the brush border surface [47]. Food matrix could positively or negatively affect bioaccessibility, consequently affecting bioavailability, highlighting the importance of performing studies on the composition and bioactivity of the digestion product.

The α-glucosidase activity of mandarin extracts of the present study showed higher IC_50_ values to those reported for other citrus samples (Table 6). For example, IC_50_ values of α-glucosidase and α-amylase inhibition from *Poncirus trifoliate* juice (a type of bitter orange) were 81.27 and 138.14 µg/mL, respectively (acarbose 35.5 ± 1.2 and 50.0 ± 0.9 µg/mL, respectively). Also, constituents of mandarin extracts (according to HPLC-DAD-MS results) have shown low IC_50_ values for narirutin, poncirin, didymin, naringin, hesperidin, and neoeriocitrin [48].

When comparing with other citrus studies, the α-glucosidase inhibition capacity of *Citrus × clementina* Hort. juice from hill and coastal plain juice were reported as higher than the ones in the present work (77.79 and 93.31 μg/mL IC_50_ values, respectively) as well as the α-amylase inhibition capacity (226.69 and 243.24 μg/mL, respectively) [49]. Particularly for extracts, α-amylase and α-glucosidase inhibition values from *Citrus medica* L. cv Diamante peel extract were 258.7 and 263.2 µg/mL (IC_50_ values), respectively (acarbose, 50.0 and 35.5 µg/mL; hesperetin, 150 and 7 µM IC_50_ values, respectively) [50]. *Citrus grandis* L. Osbeck peel extracts showed IC_50_ values of 80.77 µg/mL and 3.59 mg/mL for α-amylase and α-glucosidase inhibition activities, respectively [51]. *Citrus aurantium* peel extracts also inhibited α-glucosidase and α-amylase when measured at specific concentrations (166 and 332 µg/mL; 0.5 and 1 mg/mL, respectively) [52]. Data supported higher inhibition capacity than Clemenule and Ortanique extracts for all the samples. It is worth noting that the IC_50_ values reported in other works were obtained by different methodologies to the ones used in the present work, making it difficult to compare.

Regarding the presence of carbohydrates’ inhibitors in the intestinal fraction (Table 6), the results showed that in most cases, the digestion process decreased the inhibition capacity (higher IC_50_ values), except for Clemenule pomace and extract (α-amylase inhibition), which showed no significant differences before and after digestion (*p* > 0.05). Bioaccessibility studies on sweet orange (*Citrus sinensis*) have shown the degradation of phenolic compounds after digestion but with bioaccessible phenolic compounds after intestinal phase, mainly flavanones [29]. Ortanique pomace showed better α-glucosidase inhibition capacity than Clemenule pomace. This inhibition may be attributed to the phenolic composition of Ortanique pomace and extract with a higher proportion of isosinensetin/sinensetin/tangeretin compared to the ones of Clemenule, respectively. Moreover, the α-amylase inhibition capacity of Clemenule pomace was enhanced by in vitro gastrointestinal digestion, which may be attributed to the enhanced release of bioactive compounds. The inhibitory effect of the samples may be ascribed to the phenolic compounds’ interaction with amino acids that is present in the active site of the enzyme [53]. To the best of our knowledge, this is the first time that studies in composition and bioactivity regarding antidiabetic properties of extract digests from mandarin pomace are performed.

AGE formation results (Table 6) showed the lowest IC_50_ values (highest inhibition capacity) for Ortanique and Clemenule hydro-alcoholic-acid pomace extracts when compared with its pomace, presenting a better inhibition capacity for Clemenule pomace (*p* < 0.05). Citrus standards were also tested (naringenin IC_50_ = 0.14 ± 0.01 mg/mL, naringin IC_50_ = 1.97 ± 0.19 mg/mL, and hesperetin IC_50_ = 0.57 ± 0.04 mg/mL), showing that naringenin had the best inhibition capacity. Hesperetin and naringin followed naringenin. Compared to the extracts, naringin presented a low inhibition capacity. These results seem to indicate that naringenin and hesperetin may have been responsible for the exerted capacity by the hydro-alcoholic-acid extracts. In vitro gastrointestinal digestion showed a higher inhibition capacity for the extracts with no significant differences between Clemenule and Ortanique extracts (*p* > 0.05). To the best of our knowledge, this is the first report of the anti-glycantive capacity of citrus pomaces and extracts, the intestinal fraction of the latter, and citrus flavonoids.

Chronic high levels of blood glucose have been associated with AGE formation and thus elevated levels of glycosylated hemoglobin (HMGB1). AGE formation has also been associated with inflammation response from macrophages that release RAGE ligands [54]. Molecules with AGE inhibition and anti-inflammatory capacities may represent good candidates for the prevention/treatment of diabetic complications and degenerative diseases [54]. Thus, mandarin pomace due to its potential to modulate the formation of AGEs, NO, and ROS may have potential as a prophylactic and therapeutic supplement for chronic diseases such as diabetes, involving the disruption of carbohydrate metabolism and inflammation.

#### 3.3.3. Hypolipidemic Effect of Mandarin Pomace and Their Respective Extracts

**Figure 3:**
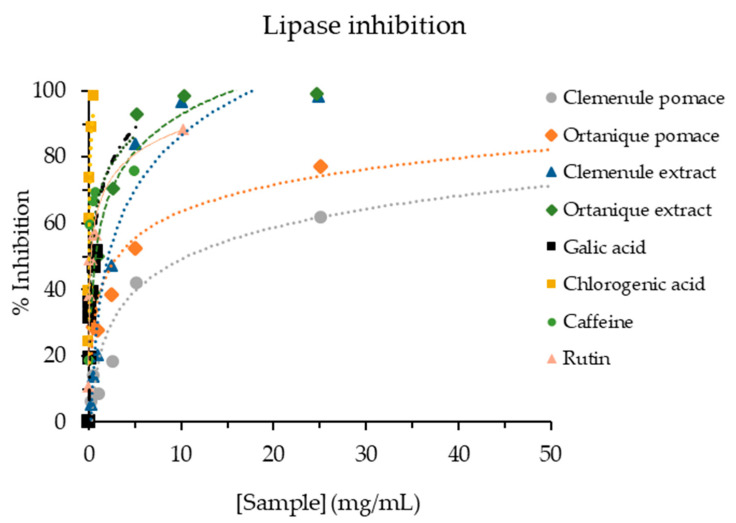
Pancreatic lipase inhibition capacity of mandarin pomaces and their respective extracts, as well as standards of phenolic compounds.

Pancreatic lipase is responsible for hydrolyzing triglycerides into fatty acids, which can be absorbed. Therefore, inhibiting this enzyme is considered a strategy for treating obesity in developed countries [45]. Clemenule and Ortanique extracts (2.259 ± 0.267 and 0.713 ± 0.065 mg/mL, respectively) showed higher pancreatic lipase inhibition than pomaces (9.740 ± 1.884 and 4.335 ± 0.610 mg/mL, respectively) (Figure 3). Ortanique pomace showed a better lipase inhibition capacity than Clemenule pomace, with the same tendency maintained for the extracts. In contrast, a Tannat grape skin’s hydro-alcoholic-acid extract such as the ones in the present work, showed similar values (2.4310 ± 0.0799 mg/mL) to that of Clemenule extract [20]. A study on citrus peel extracts showed IC_50_ values of pancreatic lipase inhibition in a range of 0.087–0.292 mg/mL (IC_50_ orlistat = 0.103 µg/mL) [55], presenting higher inhibition than the mandarin extracts of the current work.

In vivo studies have shown that oral supplementation of hesperidin regulates lipid and carbohydrate metabolism by reducing blood cholesterol and plasma insulin levels, along with exhibiting anti-hyperglycemic and hypolipidemic effects in male Wister rats with STZ-induced diabetic myocardial infarction [56]. Additionally, hesperidin and naringin have also shown to mitigate the production of pro-inflammatory cytokines (IL-6 and TNF-α) after daily administration for 4 weeks (oral dose of 50 mg/kg) to high fat-fed/streptozotocin-induced type 2 diabetic rats, while also enhancing the antioxidant defense system [57]. Furthermore, citrus peel bioflavonoids have demonstrated a modulation of lipid and glycemic profiles in experimental rodent models [58]. The reduction in visceral fat pad weight, body weight gain, and plasma triglyceride levels has been documented for polymethoxyflavanones, such as nobiletin and tangeretin from citrus peel, in high-fat diet-induced obese mice compared to the control group [59].

#### 3.3.4. Anti-Inflammatory Effect of Mandarin Pomace Extracts

**Figure 4:**
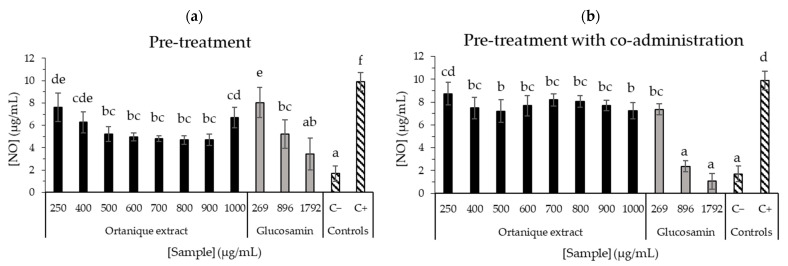
Effects of Ortanique extract, glucosamine, and positive (C+) and negative (C−) controls on LPS-induced (1 µg/mL) NO oxide production in RAW264.7 cells in the pre-treatment (**a**) and pre-treatment with co-administration (**b**) assays. Glucosamin was used as an anti-inflammatory agent of reference. They were incubated with samples for 24 h, and afterwards, samples were taken out in order to add LPS 1 µg/mL (pre-treatment) or sample with LPS 1 µg/mL (pre-treatment with co-administration) for 24 h. Supernatants were transferred to a new 96-well plate to react with Griess reagent through a 15 min incubation (room temperature in the dark) and absorbance was measured at 550 nm. NO standard curve was measured in parallel. Bars represent the mean values in NO concentration and error bars represent the standard deviation. Statistical analysis was carried out between the values in the same assay, indicating significant differences by different letters (Tukey test, *p* < 0.05). Results were compiled from triplicate measurements from three different cell passages.

**Figure 5:**
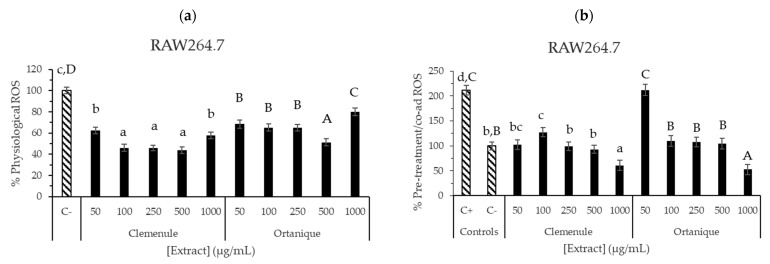
ROS production inhibition by Ortanique and Clemenule extracts tested in RAW264.7 macrophages. Physiological (**a**) and induced (**b**) % ROS were determined by 24 h incubation with samples (physiological (**a**)), followed by 30 minutes of incubation with samples and t-BOOH (pre-treatment with co-administration (**b**)). Bars and error bars represent the mean values and standard errors, respectively. Lowercase letters indicate significant differences between the samples that present them (LSD Fisher, *p* < 0.05). Capital letters indicate significant differences between the samples that present them (LSD Fisher, *p* < 0.05). Results were compiled from triplicate measurements from three different cell passages.

Regarding anti-inflammatory capacity (Figure 4 and Figure 5), Ortanique extract showed a significant reduction in nitric oxide (NO) production in RAW264.7 macrophages in the pre-treatment assay at all the tested concentrations (250–1000 µg/mL), observing a pro-inflammatory effect at 1000 µg/mL by a mild increase. In the pre-treatment assay, the concentrations presented a dose-dependent like behavior as they showed significant differences by LSD Fisher between 250, 400 and the rest of the concentrations (500–900 µg/mL, *p* > 0.05 in this range), with a pro-inflammatory effect on a concentration of 1000 µg/mL (*p* < 0.05). By contrast, Clemenule extract did not reduce NO formation. Ortanique extract concentrations from 500 to 900 µg/mL inhibited the formation of NO, without significant differences (*p* > 0.05) with glucosamine at 896 µg/mL (5 mM), which is an anti-inflammatory agent of reference. With regard to the pre-treatment with co-administration assay, the results suggest that Ortanique extract presents a very low capacity of inhibiting NO formation at concentrations of 400–1000 µg/mL compared to glucosamine at 5 and 10 mM (896 and 1792 µg/mL, respectively) as the reference anti-inflammatory agent. Glucosamine presented IC_50_ values of 1.192 and 0.645 mg/mL in pre-treatment and pre-treatment with co-administration assays, respectively.

ROS formation after the extracts’ treatment was also assessed in RAW264.7 cells, finding physiological ROS inhibition when compared to negative control (C−, *p* < 0.05) (Figure 5a,b). Ortanique extract showed inhibition up to 500 µg/mL (1–500 µg/mL), and an increase at 1000 µg/mL, with no significant differences with negative control (*p* > 0.05), thus showing a pro-oxidant effect. Clemenule extract exerted inhibition at all the concentrations tested (1–1000 µg/mL). Ascorbic acid was also assessed, showing a high inhibition of physiological ROS formation. The pre-treatment assay was also performed, where the lowest concentrations of Ortanique (1–25 µg/mL) and Clemenule (1 µg/mL) extracts were the only ones showing ROS inhibition formation when compared to the positive control (*p* < 0.05). Pre-treatment with co-administration assay showed inhibitory activity for both extracts, except at 50 µg/mL of the Ortanique extract, finding the highest inhibition of intracellular ROS formation at a concentration of 1000 µg/mL. These results suggest the antioxidant potential of Clemenule and Ortanique extracts in counteracting intracellular ROS formation on murine macrophages (RAW264.7) under physiological and induced conditions. ROS are naturally produced by immune system cells such as macrophages and neutrophils during inflammation through the release of cytokines that recruit more of these cells. Therefore, reducing ROS formation is crucial for ameliorating inflammation [8]. Particularly, the reduction in ROS formation by macrophages is interesting under chronic inflammation conditions, which are related to non-communicable chronic diseases such as diabetes [5].

The phenolic composition of mandarins (ferulic, coumaric, caffeic, nobiletin, hesperidin, tangeretin, and their derivatives, among others) [2] may have a key role in macrophage NO production inhibition. Ortanique extract identified flavonoids (mostly tetramethylscutellarein, nobiletin, and tangeretin) have been reported for preventing NO production [2].

In the literature, many studies have reported naringenin (among other mandarin polyphenols) as an inhibitor of ROS formation in AGE-BSA-treated RAW264.7 cells. Additionally, naringenin has been observed to inhibit the release of pro-inflammatory cytokines (TNF-α and IL-1β) under AGE-induced inflammation with MGO-BSA (60 µM/mL) [60].

In agreement with the results of the current research, Nakajima et al. [61] found a significant decrease in NO production in RAW264.7 macrophages when stimulated with LPS via all the tested citrus residue extracts at a concentration of 1.00 mg/mL, and at a dose of 0.2 mg/mL only through the biotransformed citrus residue extract. Ho and Kuo [62] investigated the anti-neuroinflammatory activity of several compounds (including nobiletin, hesperidin, narirutin, naringin, neohesperidin, and tangeretin), using an LPS-induced BV2 microglia model. In their work, NO production was inhibited (25–100 µM) with the exception of tangeretin, which showed lower potency. Notably, nobiletin demonstrated the most significant inhibition of NO production among the tested compounds. Particularly, nobiletin has been reported as a useful agent for ameliorating inflammation in RAW264.7 macrophages under LPS-induced conditions [5], being the main flavonoid present in Clemenule and Ortanique extracts. Also, the stimulation of lipolysis and triglyceride accumulation decrease in adipocytes was reported for orange peel extracts [63]. All these reports suggest that flavonoids may be responsible for the exerted anti-inflammatory capacity by Ortanique and Clemenule extracts.

All the previous reports and the present work on citrus byproducts’ anti-inflammatory capacity re-enforce the potential of these byproducts as functional ingredients through the control of systemic and low-grade chronic inflammation promoted by oxidative stress [5]. However, in vivo studies should be carried out in order to confirm the extracts’ effects.

**Figure 6:**
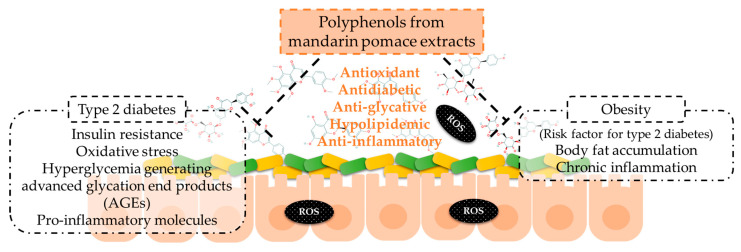
Mechanisms of action of the phenolic compounds of mandarin pomace extracts to counteract type 2 diabetes and obesity.

In summary, the results of the present work are preliminary results on mandarin pomace extracts’ effects on oxidative stress and inflammation, as well as on energetic metabolism (carbohydrates and lipids) and AGE formation by the confirmation of phenolic compounds after in vitro digestion. This hypothesis should be confirmed by in vivo assays. The results support the action of phenolic compounds composing mandarin extracts that remain after the digestion process on the pathogenesis of non-communicable chronic diseases (Figure 6). Specifically, these effects are associated with type 2 diabetes and obesity pathogenesis, which include these points, and the diet is a key factor for their prevention and reduction of symptoms [44]. It is stated that obesity is a risk factor of type 2 diabetes, so its prevention may play a role in the incidence of the latter [13]. Moreover, our preliminary results show the action of phenolic compounds to counteract oxidative stress at the intestinal level, showing promising potential for enhancing intestinal health.

## 4. Conclusions

Our results showed that hydro-alcoholic-acid extracts from Clemenule and Ortanique pomace presented antioxidant, anti-inflammatory, antidiabetic, anti-glycative, and hypolipidemic potential, probably at least partially because of their composition in flavonoids. After in vitro simulation of digestion, the extracts also showed carbohydrates’ bioaccessibility and antioxidant, antidiabetic, and anti-glycative potential, probably as a result of the action of the phenolic compounds remaining in the intestinal fraction. Nobiletin was the major flavonoid found in both extracts, and other main flavonoids such as tangeretin, heptamethoxyflavone, tetramethylscutellarein, hesperidin/neohesperidin, and naringin/narirutin were also found. Moreover, nobiletin was the main flavonoid detected in the intestinal fraction of Clemenule pomace and its extract, in contrast with Ortanique samples that presented isosinensetin/sinensetin/tangeretin as the major compounds.

In addition, the extracts inhibited intracellular ROS formation on CCD-18Co colon cells under tert-butyl hydroperoxide (1 mM)-induced conditions. Moreover, Ortanique extract presented anti-inflammatory potential, which was confirmed by a decreased production of nitric oxide in RAW264.7 macrophages under LPS-induced conditions. The extracts also inhibited intracellular ROS formation in RAW264.7 macrophages under induced conditions. The analyses of composition and bioactivity of the digested samples supported the potential of these extracts as functional ingredients associated with the remaining bioactive compounds after the physiological process. Altogether, the preliminary results obtained so far suggest that hydro-alcoholic-acid extracts from mandarin pomace have the potential to reduce key events associated with the pathogenesis of chronic diseases, including diabetes. The effects may be at least partially associated with the presence of phenolic compounds. In vivo studies are needed for establishing a cause–effect relationship and the effective dose for achieving this goal in humans.

## Figures and Tables

**Table 1:** Total carbohydrates, sucrose, glucose, and fructose contents of mandarin pomaces, extracts, and wastes of the extraction process (insoluble fractions).

Sample	Total Carbohydrates	Sucrose	Glucose	Fructose
Clemenule	35.65 ± 1.71 ^b^	4.39 ± 1.50 ^a^	9.34 ± 1.26 ^a^	15.28 ± 0.71 ^a^
Ortanique	34.57 ± 5.68 ^b^	5.10 ± 0.81 ^a^	11.15 ± 0.99 ^a^	15.95 ± 1.27 ^a^
Clemenule extract	65.77 ± 5.56 ^d^	34.22 ± 2.49 ^c^	7.36 ± 3.45 ^a^	35.66 ± 7.01 ^b^
Ortanique extract	53.06 ± 4.68 ^c^	24.27 ± 1.99 ^b^	16.99 ± 1.44 ^b^	37.68 ± 4.55 ^b^
Clemenule insoluble fraction	6.89 ± 0.53 ^a^	-	^-^	-
Ortanique insoluble fraction	6.88 ± 0.57 ^a^	-	^-^	-

Results are expressed as means ± SD (*n* = 6) (g/100 g of dry sample). Different letters mean significant differences by the Tukey test (*p* < 0.05).

**Table 2:** Sucrose, glucose, and fructose contents in digests (intestinal fractions) of Clemenule and Ortanique mandarin pomaces and the corresponding extracts.

Sample	Sucrose	Glucose	Fructose
Clemenule pomace	3.80 ± 0.35 ^a^	3.70 ± 1.65 ^a^	10.42 ± 0.61 ^a^
Ortanique pomace	3.55 ± 0.80 ^a^	3.83 ± 0.30 ^a^	10.17 ± 0.38 ^a^
Clemenule extract	8.44 ± 1.12 ^b^	3.81 ± 0.85 ^a^	15.27 ± 0.65 ^b^
Ortanique extract	10.16 ± 1.82 ^b^	5.46 ± 2.09 ^a^	17.29 ± 3.39 ^b^

Results are expressed as means ± SD (*n* = 6) (g/100 g of dry sample). Different letters mean significant differences by Tukey test (*p <* 0.05).

**Table 3:** Phenolic compound identification of Clemenule and Ortanique extracts by UHPLC-DAD-MS/MS.

**Positive ESI**					
Compound ^1^	Clemenule extract ^2^	Ortanique extract ^2^	RT (min)	[M+H]^+^ (*m*/*z*)	Fragments (*m*/*z*)
Rhoifolin/Isorhoifolin	0.000999	0.001663	11.0	579.1708	271.0595
Isosinensetin/Sinensetin/Tangeretin 1	0.016582	0.026516	16.2	373.1282	343.0806, 153.0181
Isosinensetin/Sinensetin/Tangeretin 2	0.039204	0.081296	16.7	373.1282	343.0806, 153.0181
Nobiletin	0.097034	0.169946	17.3	403.1387	373.091, 183.0288
Heptamethoxyflavone	0.089591	0.019377	17.6	433.1493	403.1019, 418.1251
Tetramethylscutellarein	0.040104	0.143404	17.7	343.1176	313.0701, 153.0180
Isosinensetin/Sinensetin/Tangeretin 3	0.044997	0.134339	18.1	373.1282	343.0806, 153.0181
**Negative ESI**					
Compound ^1^	Clemenule extract ^2^	Ortanique extract ^2^	RT (min)	[M-H]^−^ (*m*/*z*)	Fragments (*m*/*z*)
Nariturin-4-glucoside/Naringin glucoside	0.000068	0.000353	9.6	741.2248	271.0639, 151.0035
Rutin	0.004279	0.005631	10.3	609.1461	301.0350, 271.0257
Eriocitrin/Neoeriocitrin 1	0.001618	n.d.	10.6	595.1668	287.0580, 151.0034
Naringin/Narirutin	0.008252	0.026907	11.2	579.1719	271.0637, 151.0035
Diosmin isomer 1	0.000730	0.001610	11.3	607.1668	299.0580, 284.0338
Eriocitrin/Neoeriocitrin 2	0.000162	0.000044	11.3	595.1668	287.0580, 151.0034
Diosmin isomer 2	0.000072	0.000175	11.4	607.1668	299.0580, 284.0338
Hesperidin/ Neohesperidin	0.037499	0.027973	11.6	609.1825	301.0739, 151.0035
Poncirin/Isosakuranetin-7-O-rutinoside	0.000177	0.002077	13.1	593.1876	285.0763
TIC	1,001,915,855	1,050,566,219			

n.d.: Not detected; ^1^ Compound Discoverer 3.1 (mzCloud library, Advanced Mass Spectral Database); ^2^ results normalized with TIC area (area/area TIC).

**Table 4:** Phenolic compound identified in digests of Clemenule and Ortanique pomaces and extracts by HPLC-DAD-MS.

		Intestinal Fraction				Colonic Fraction	
Compound	[M+H]^+^	O	C	EO	EC	O	C
Isosinensetin/Sinensetin/Tangeretin 1	373	18,176	8145	122,121	9009	10,265	7488
Isosinensetin/Sinensetin/Tangeretin 2	373		531		122,276	898	117
Nobiletin	403	662	110,244	2187	488,478	238	254
Heptamethoxyflavone	433	4625	28,971	7865	7245	933	3474
Isosinensetin/Sinensetin/Tangeretin 3	373	807	220	22,460	899	22,481	341
Total area		24,932	148,111	154,633	627,907	34,815	11,674

O: Ortanique pomace; C: Clemenule pomace; EO: Ortanique extract; EC: Clemenule extract.

**Table 5:** Results of overall antioxidant capacity (Folin, ABTS, and ORAC-FL methods) of Clemenule and Ortanique mandarin pomaces, extracts, and their respective digests (intestinal fractions), as well as insoluble fractions of pomace extraction (waste of the extraction process).

Samples	Folin Reaction (mg GAE/g Sample)		ABTS (µmol TE/g Sample)		ORAC-FL (µmol TE/g Sample)	
	Samples	Digests	Samples	Digests	Samples	Digests
Clemenule	17.73 ± 0.75 ^d^	9.98 ± 0.51 ^bc^	108.4 ± 2.1 ^c^	90.1 ± 1.5 ^b^	284.6 ± 14.4 ^cd^	135.5 ± 22.2 ^a^
Clemenule extract	26.86 ± 3.47 ^f^	9.74 ± 0.76 ^bc^	310.6 ± 9.6 ^f^	83.2 ± 2.6 ^b^	346.7 ± 34.7 ^d^	142.8 ± 38.1 ^ab^
Clemenule insoluble fraction	6.93 ± 0.20 ^a^	-	51.1 ± 1.3 ^a^	-	587.4 ± 61.1 ^f^	-
Ortanique	16.72 ± 0.35 ^d^	11.00 ± 0.65 ^c^	120.8 ± 4.1 ^d^	92.0 ± 2.0 ^b^	288.2 ± 24.0 ^cd^	232.5 ± 26.7 ^bc^
Ortanique extract	22.29 ± 3.19 ^e^	9.20 ± 0.58 ^abc^	271.7 ± 13.0 ^e^	91.7 ± 10.3 ^b^	269.0 ± 43.3 ^cd^	891.6 ± 123.8 ^g^
Ortanique insoluble fraction	7.75 ± 0.31 ^ab^	-	63.2 ± 1.7 ^a^	-	483.4 ± 24.8 ^e^	-

Results are expressed as means ± SD (n = 3). Statistical analysis was carried out between the values in the same assay, indicating significant differences by different letters (Tukey test, *p* < 0.05).

**Table 6:** α-glucosidase and α-amylase, and fluorescent AGE formation inhibition capacities (IC_50_ values, half maximal inhibitory concentration) of pomaces, extracts, their respective digests, and standards.

Samples	IC_50_ (mg/mL)					
	α-Glucosidase		α-Amylase		AGE Inhibition	
	Samples	Digests	Samples	Digests	Samples	Digests
Clemenule	4.92 ± 0.27 ^d^	3.97 ± 0.97 ^c,d^	70.19 ± 11.16 ^c^	58.04 ± 2.09 ^b,c^	3.25 ± 0.47 ^b^	31.23 ± 2.57 ^c^
Clemenule extract	2.08 ± 0.08 ^a,b^	13.50 ± 0.37 ^f^	16.23 ± 0.19 ^a^	28.79 ± 1.33 ^a^	1.00 ± 0.05 ^a^	16.50 ± 1.38 ^ab^
Ortanique	3.42 ± 0.64 ^b,c^	4.93 ± 0.41 ^d^	50.07 ± 2.42 ^b^	105.68 ± 16.03 ^d^	5.37 ± 0.20 ^c^	18.60 ± 3.91 ^b^
Ortanique extract	1.72 ± 0.09 ^a^	11.07 ± 1.11 ^e^	19.15 ± 0.18 ^a^	69.64 ± 1.22 ^c^	1.19 ± 0.08 ^a^	12.77 ± 0.29 ^a^
Standards (µg/mL)	AC = 4.0 ± 0.3		AC = 34.1 ± 0.8		AG = 46.5 ± 2.8	

Results are expressed as means ± SD (n = 3). Statistical analysis was carried out between the values in the same assay (samples and digests), indicating significant differences by different letters (Tukey test, *p* < 0.05). All standards presented significant differences with the samples in the same assay (standards were excluded from the statistical analysis displayed in the table for a better appreciation of significant differences). AC: acarbose; AG: aminoguanidine.

## Data Availability

The original contributions presented in the study are included in the article/Supplementary Materials; further inquiries can be directed to the corresponding author.

## Supplementary Materials

The following supporting information can be downloaded at: https://www.mdpi.com/article/10.3390/nu16142370/s1, Figure S1: Cell viability of RAW264.7 and CCD-18Co cells by MTT assay. EC (Clemenule extract), EO (Ortanique extract), and controls were tested. Negative control (C−) consisted of medium without FBS, positive control (DMSO) consisted of DMSO 50% in medium without FBS, and ascorbic acid (Asc) in a concentration of 10 µg/mL. Bars and error bars represent the mean values and standard deviation, respectively. Different letters state significant differences by Tukey test (*p* < 0.05). All determinations were performed in triplicate in three different cell passages.
