# Supplementary material for: Health-Promoting Potential of Mandarin Pomace Extracts Enriched with Phenolic Compounds
Source: Nutrients. 2024 Jul 22;16(14):2370. doi: 10.3390/nu16142370 (PMC11280356; doi:10.3390/nu16142370)
Supplement: Supplementary file 1 [file nutrients-16-02370-s001.zip › nutrients-3087154-supplementary.pdf]

Cell viability (MTT assay)

Nighty six-well plates were used seeding 10.000 CCD-18Co and 80.000 RAW264.7 cells/well. After seeding, the cells were incubated in the same conditions as stated above for 24 h, till cell-confluence was achieved. Then, cells were incubated with different concentrations of the extracts (1, 10, 25, 50, 100, 250, 500 and 1000 μg/mL) for 24 hours (150 μL). When time was completed, 20 μL of MTT (6 mM) reagent was added to each well of the 96-well plate and, the plate with CCD-18Co cells was incubated for 3 hours and the plate with RAW264.7 cells was incubated for 30 minutes. After the incubation, supernatants were removed from the cells and 100 μL of DMSO was added to each well, followed by 5 minutes' incubation time allowing better homogenization, and absorbance at 570 nm was measured. Viability percentage was calculated by taking the absorbance value of the control (DMEM) as 100 %.

Prior to the analysis of antioxidant and anti-inflammatory properties, cell viability on CCD-18Co and RAW 264.7 cells was assessed at different concentrations of Clemenule and Ortanique extracts (EC and EO, respectively) (**Figure S1**). The viability for CCD-18Co and RAW 264.7 cells was greater than 80% of the negative control (C-) for the tested concentrations of the extracts as well as for ascorbic acid (Asc) on CCD18-Co cells. Nevertheless, the highest concentrations (from 500 μg/mL) of the extracts represented a greater decrease on RAW 264.7 cells viability than on CCD-18Co cells. As to RAW264.7 cells, Nakajima et al. [61] found that the cell viability of RAW264.7 murine macrophages was not affected at concentrations 0.01-1.00 mg/mL when incubated with citrus residue extracts.

A

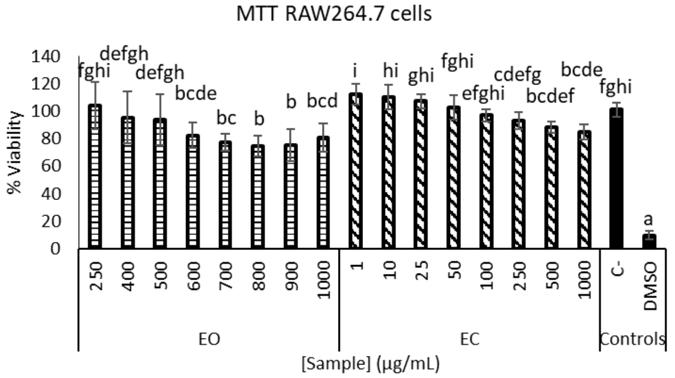


B

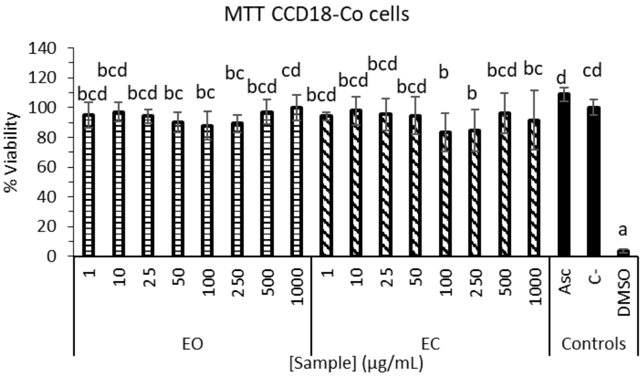


**Figure S1.** Cell viability of RAW264.7 and CCD-18Co cells by MTT assay. EC (Clemenule extract), EO (Ortanique extract) and controls were tested. Negative control (C-) consisted of medium without FBS, positive control (DMSO) consisted of DMSO 50 % in medium without FBS, and ascorbic acid (Asc) in a concentration of 10 µg/mL. Bars and error bars represent the mean values and standard deviation, respectively. Different letters state significant differences by Tukey test (*p* < *0.05*). All determinations were performed in triplicate in three different cell passages.

Nakajima, V.M.; Moala, T.; Caria, C.R. e P.; Moura, C.S.; Amaya-Farfan, J.; Gambero, A.; Macedo, G.A.; Macedo, J.A. Biotransformed Citrus Extract as a Source of Anti-Inflammatory Polyphenols: Effects in Macrophages and Adipocytes. Food Res. Int. 2017, 97, 37–44, doi:10.1016/j.foodres.2017.03.034.
